# Supplementary material for: Janus microparticles-based targeted and spatially-controlled piezoelectric neural stimulation via low-intensity focused ultrasound
Source: Nat Commun. 2024 Mar 5;15:2013. doi: 10.1038/s41467-024-46245-4 (PMC10915158; doi:10.1038/s41467-024-46245-4)
Supplement: Supplementary file 1 — Supplementary Information [file 41467_2024_46245_MOESM1_ESM.pdf]

Supplementary Material

for

Janus Microparticles-based Targeted and Spatially-Controlled Piezoelectric Neural Stimulation via Low-Intensity Focused Ultrasound

Mertcan Han[1,2], *Erdost Yildiz*[1], *Ugur Bozuyuk*[1], *Asli Aydin*[1,3], *Yan Yu*[1], *Aarushi Bhargava*[1], *Selcan Karaz*[1,2] *& Metin Sitti*[1,2,4,*]

[1] Physical Intelligence Department, Max Planck Institute for Intelligent Systems, 70569 Stuttgart, Germany

[2] Institute for Biomedical Engineering, ETH Zurich, 8092 Zurich, Switzerland

[3] Department of Neurosurgery, Maastricht University Medical Centre, Maastricht, Netherlands

[4] School of Medicine and College of Engineering, Koç University, 34450 Istanbul, Türkiye

[*] Correspondence to: sitti@is.mpg.de

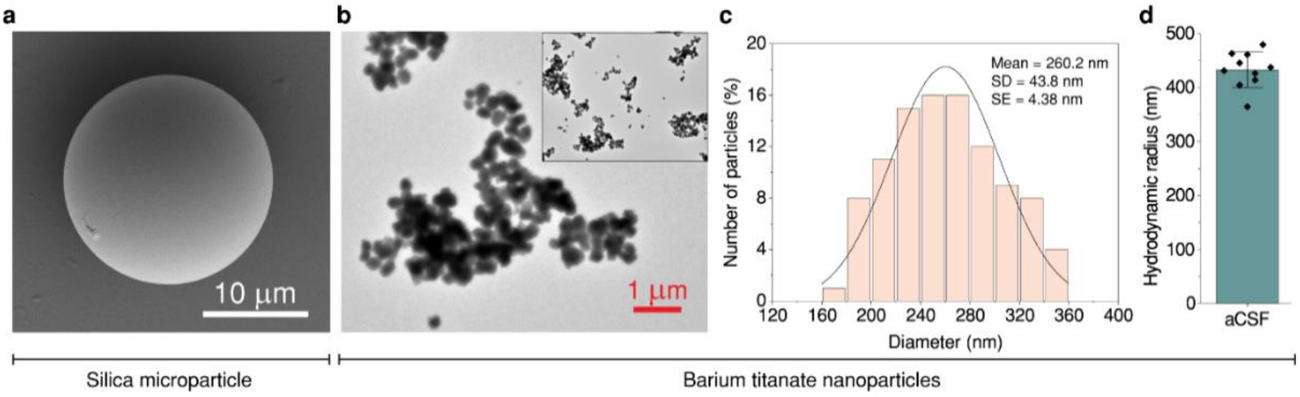

Supplementary Fig. 1 a SEM image of the unmodified silica microparticles. **b** Example TEM image of BTNPs. A larger field of view could be seen in the inset. **c** Particle size distribution analysis of three different TEM imaging experiments. **d** Dynamic light scattering (DLS) was used to characterize MENP hydrodynamic properties in artificial cerebrospinal fluid (aCSF).

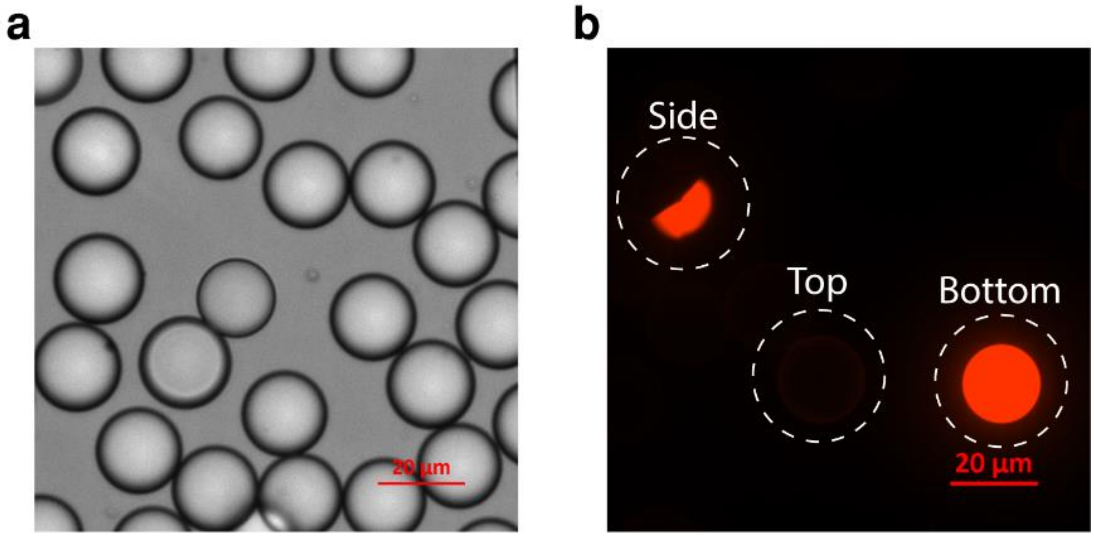

Supplementary Fig. 2 a Bright-field microscope image of the silica microparticles before the PEMP fabrication. **b** Example fluorescence image of PEMPs in three orientations indicated by dashed circles. The side view represents the condition of the magnetization direction of PEMPs in the xy plane, similarly, the magnetization direction is in +z and -z direction for top and bottom orientations, respectively.

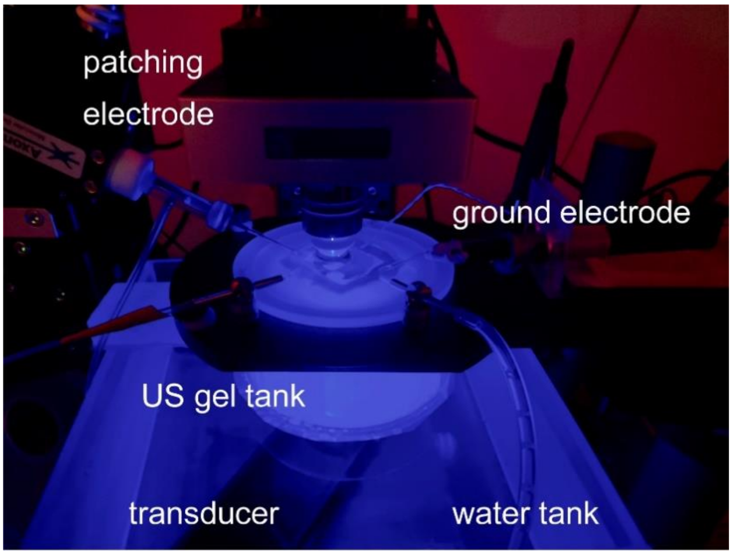

Supplementary Fig. 3 Experimental schematic for patch-clamp electrophysiology system. Photograph of the custom-built measurement system for fluorescence imaging and patch-clamp recordings on primary neurons under FUS excitation.

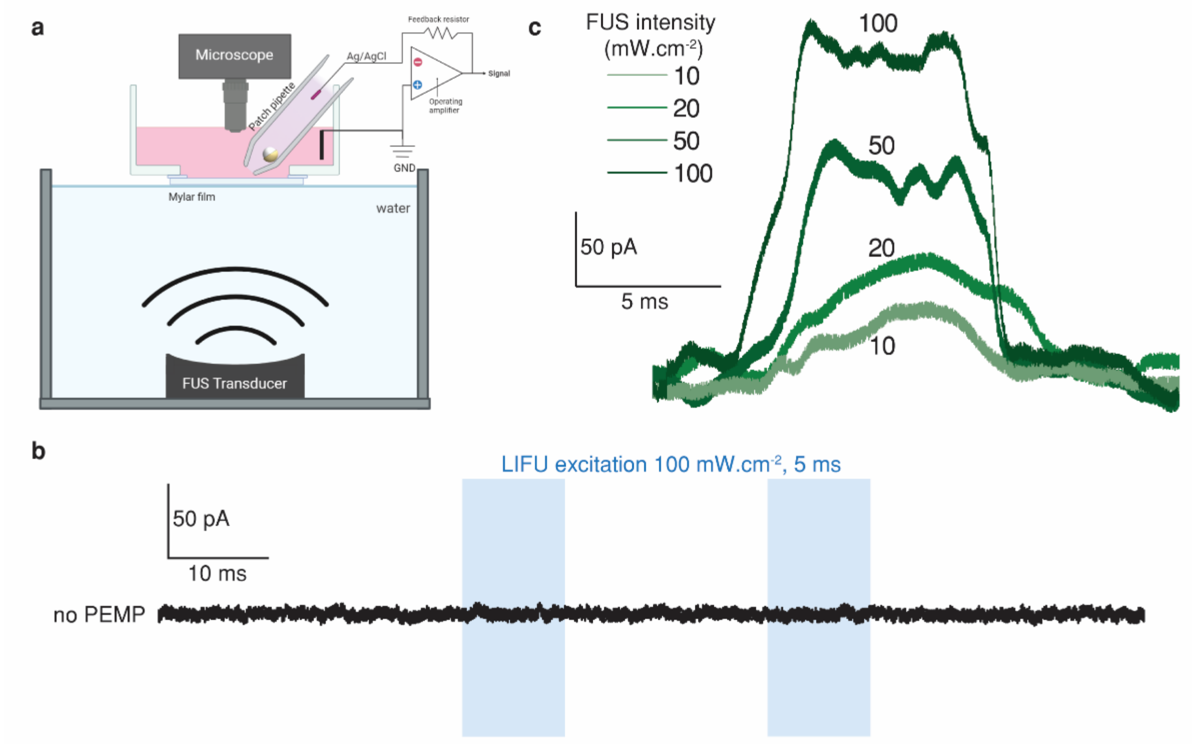

Supplementary Fig. 4 Single PEMP recordings to investigate piezoelectric current generation under FUS. a Experimental schematic of piezoelectric current measurement system. **b** Current trace from glass patch pipette with no PEMPs under two pulses of 10 ms 100 mW.cm^{-2} FUS. This is a representative trace from n = 18 measured traces. Blue rectangles represent the time points of FUS excitation. **c** Piezoelectric current traces from a single PEMP under 10 ms 10, 20, 50, and 100 mW.cm^{-2} FUS. The potential was held at zero in voltage-clamp mode.

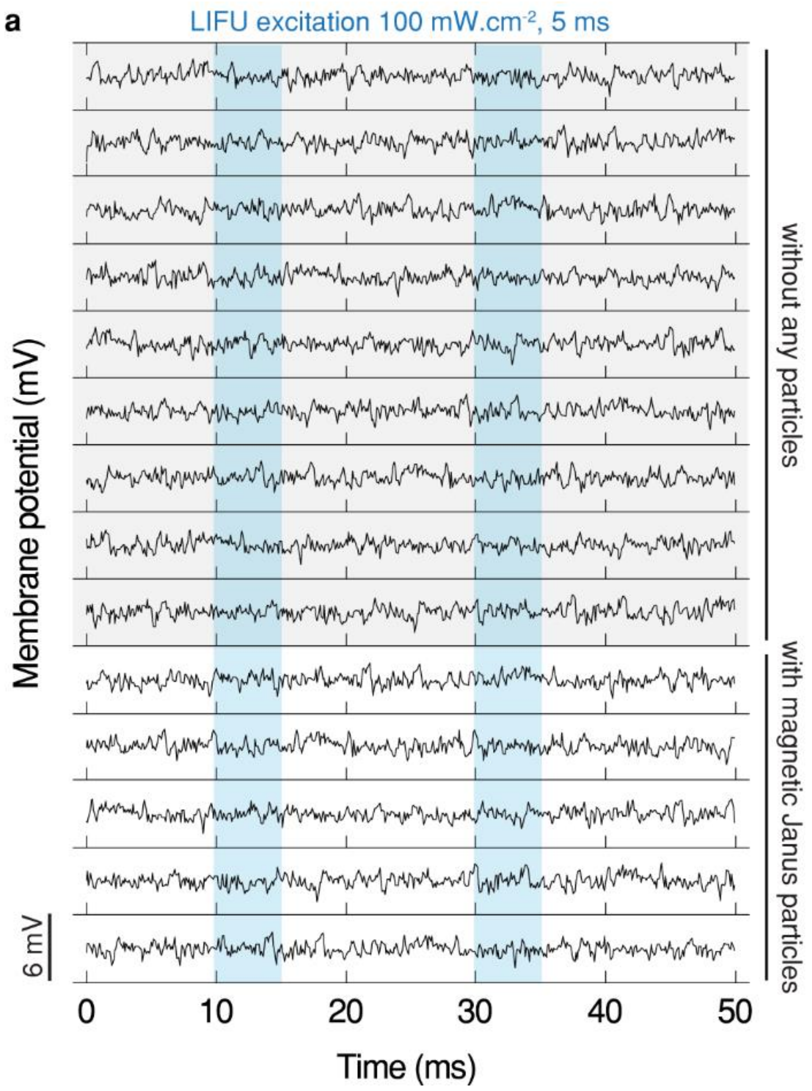

Supplementary Fig. 5 Control experiments on primary neurons under LIFU with and without magnetic Janus particles. a Representative membrane potential traces of primary neurons excited under LIFU (two pulses of 5 ms 100 mW.cm-2 FUS) with and without magnetic Janus microparticles.

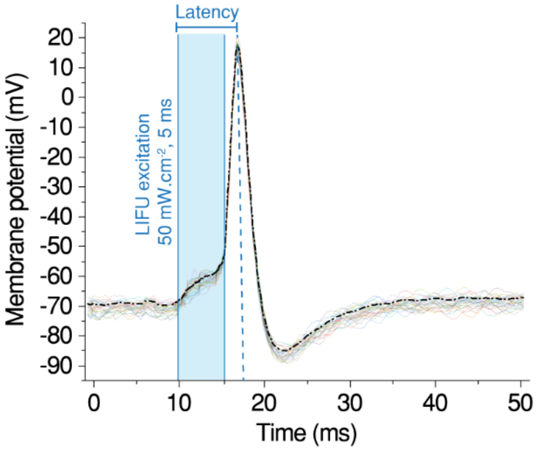

Supplementary Fig. 6 Membrane potential change during PEMP-induced stimulation. Individual patch-clamp electrophysiology recording of primary neurons excited with 100 mW.cm-2, 5 ms, 2 MHz LIFU while PEMP was located near the recorded neuron <5 µm (n = 29 pulses). The mean latency was calculated as ~7.16 ms.

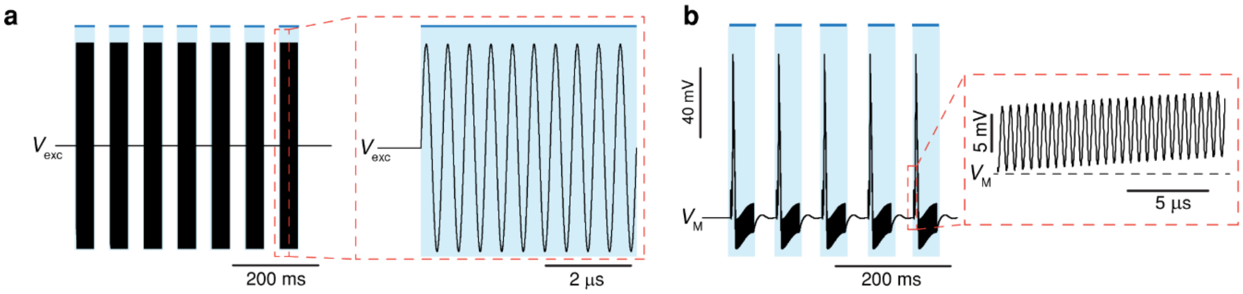

Supplementary Fig. 7 Excitation signal and the induced membrane potential change simulated using the Hodgkin–Huxley (HH) model. a FUS excitation signals with 2 MHz center frequency and 50 Hz burst frequency with 20 ms ON time. The inset shows the driving signal of 2 MHz. **b** HH model numerical simulations for transmembrane potential with driving signal depicted in a. Inset shows the depolarization window with 2 MHz FUS excitation.

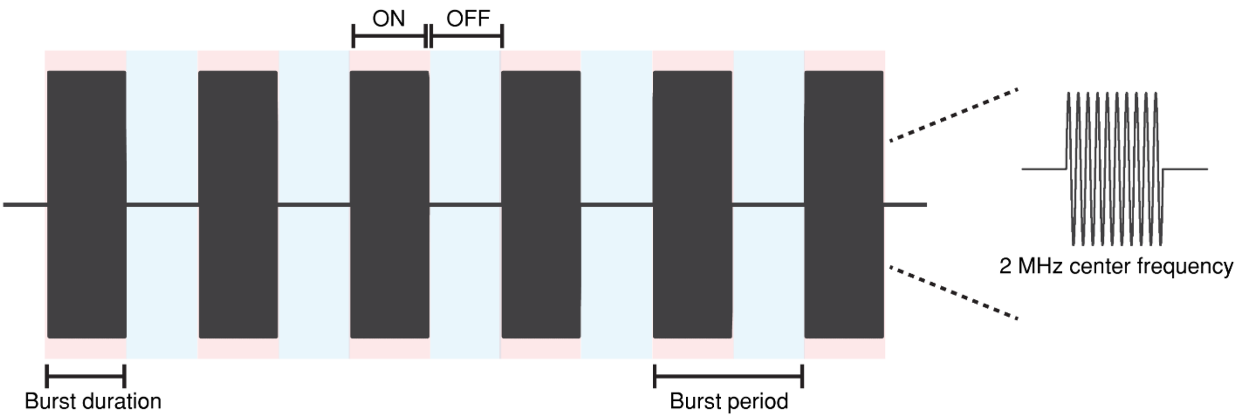

Supplementary Fig. 8 Representative waveform generated for driving FUS transducer. The center frequency of the FUS probe is 2 MHz in this study. The burst duration and period determine the desired stimulation frequency. The ON and OFF periods were the same for all experiments, indicating a 50% duty cycle for burst stimulation waveforms.

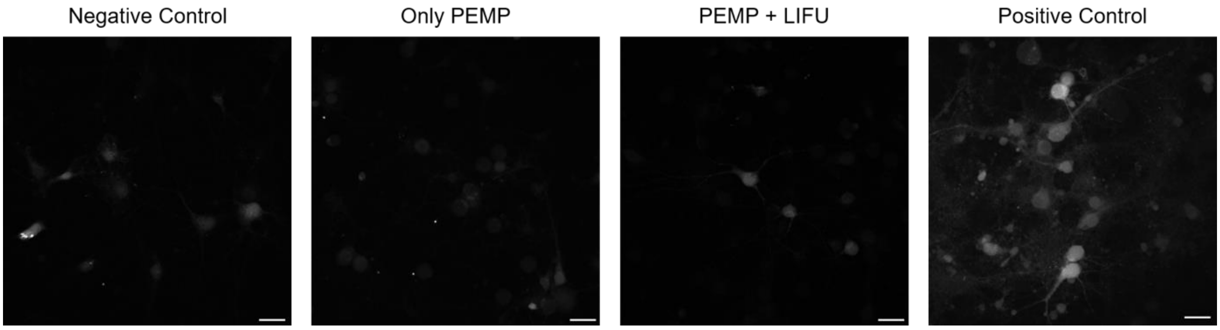

Supplementary Fig. 9 Intracellular oxidative stress measurement images with H2DCFDA staining for the primary neurons. The negative control is primary neurons without any treatment, whereas the positive control is the cells with 100 µM H2O2 treatment. Scale bar: 100 µm.

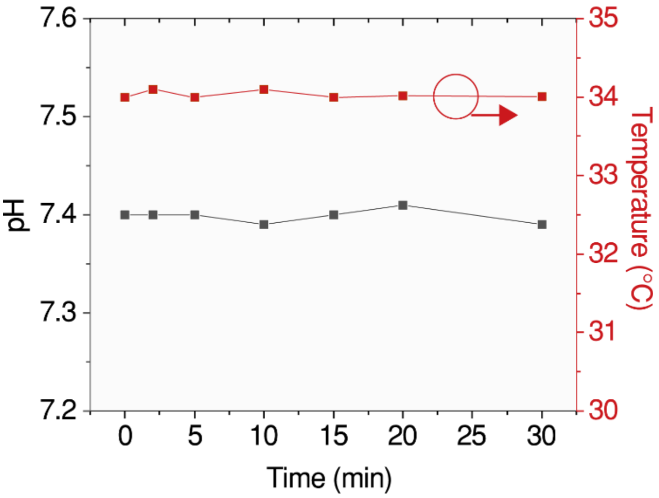

Supplementary Fig. 10 pH and temperature monitoring during neural stimulation experiments. pH and temperature recording of the patch-clamp measurement chamber to monitor any environmental change in the extracellular medium. For the pH (left, black), we took aliquots at specific time frames, while the temperature (right, red) was continuously monitored with the temperature probe.

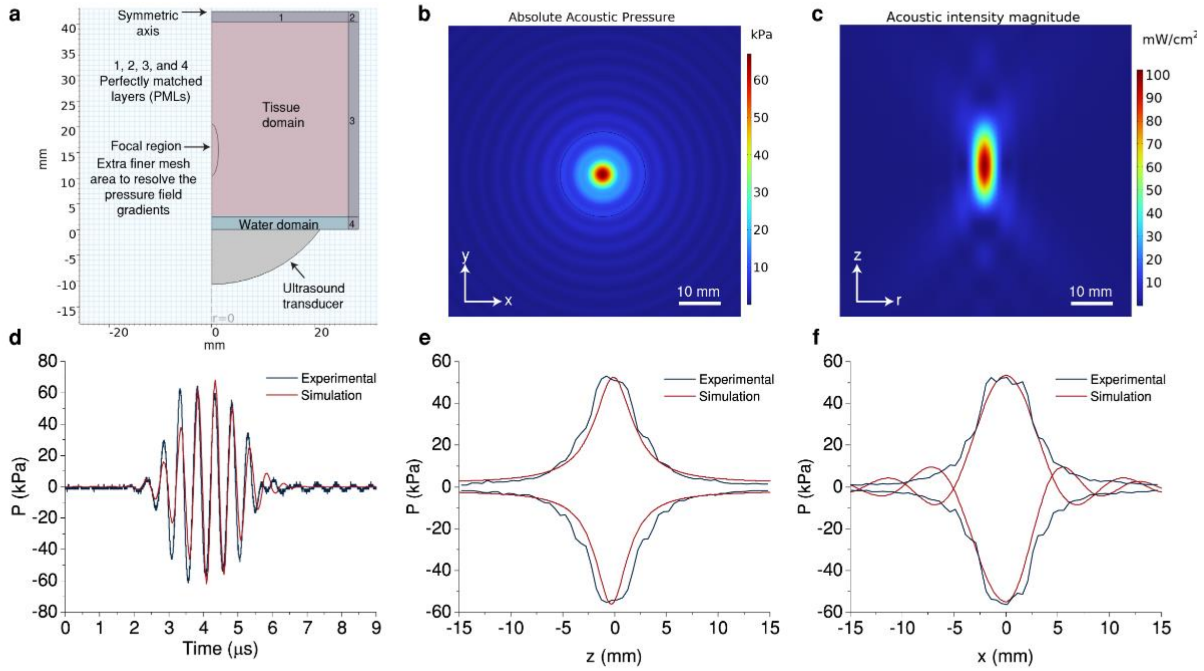

Supplementary Fig. 11 Experimental and numerical investigation of pressure waves generated by the FUS transducer. a 2D representation of ultrasound transducer/water/tissue phantom system. Model parts are marked in the figure. The model possesses the axial symmetry that enables three-dimensional reconstruction of the 2D computations. Numerical results and corresponding heat maps of **b** absolute acoustic pressure in xy plane at the center of the FUS focus and **c** acoustic intensity magnitude in zr plane. **d** Experimental and simulation results of transient pressure waves for a single burst pulse of FUS at 2 MHz. Acoustic pressure profile along **e** the symmetry axis and **f** radial direction in the focal plane.

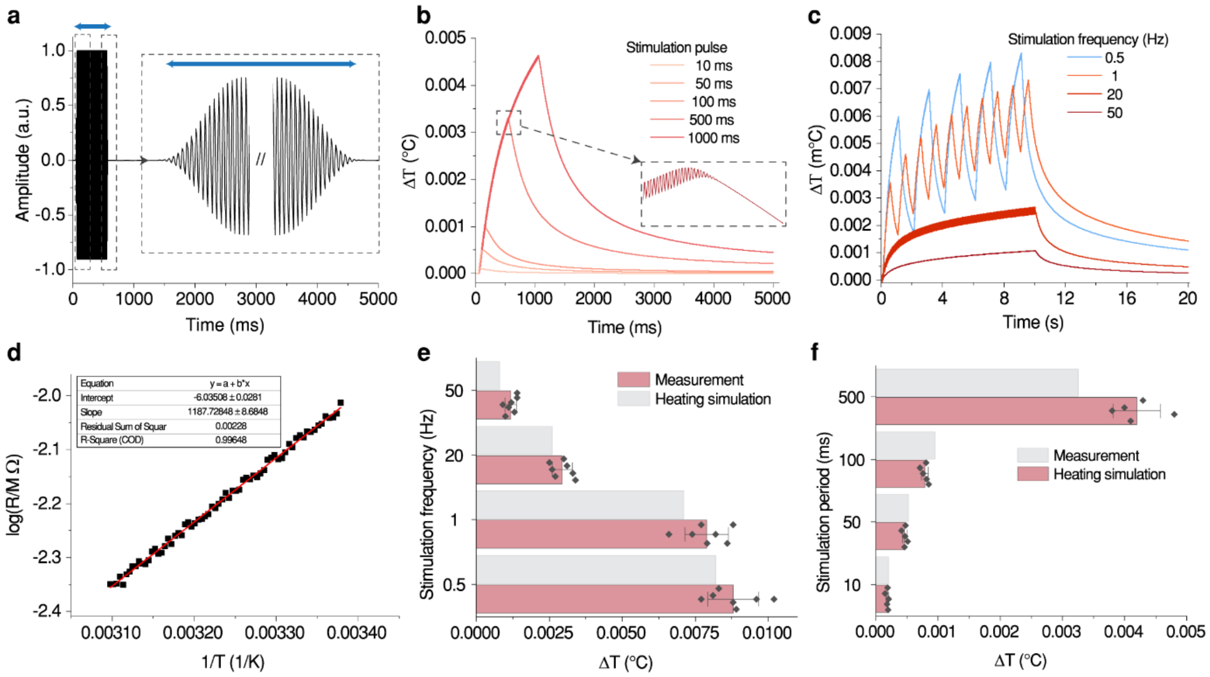

Supplementary Fig. 12 Evaluating thermal effects by numerical simulations and resistance-temperature measurements. a FUS driving signal for temperature measurements. The driving signal is at 2 MHz modulated with a single pulse with a rise and fall time of 10 µs. Inset shows the rise and fall time. **b** Heating calculations of brain phantom under continuous 100 mW.cm^{-2} FUS for changing pulse durations. The inset shows 2 MHz heating/cooling cycles. **c** Heating calculations of brain phantom under 2 MHz 100 mW.cm^{-2} FUS for changing pulse frequencies. **d** Calibration curves using a linear fit of recorded pipette resistances and corresponding extracellular medium temperatures. The linear fit shows the relationship between the pipette resistance and temperature. This calibration was utilized to measure the temperature increase under 100 mW.cm^{-2} FUS for **e** changing pulse frequency and **f** duration. The bar plots in e and f demonstrate the numerical calculations and experimental results for n = 7 and n = 5 independent experiments for pulse frequency and duration, respectively. Data are presented as mean ± s.d.

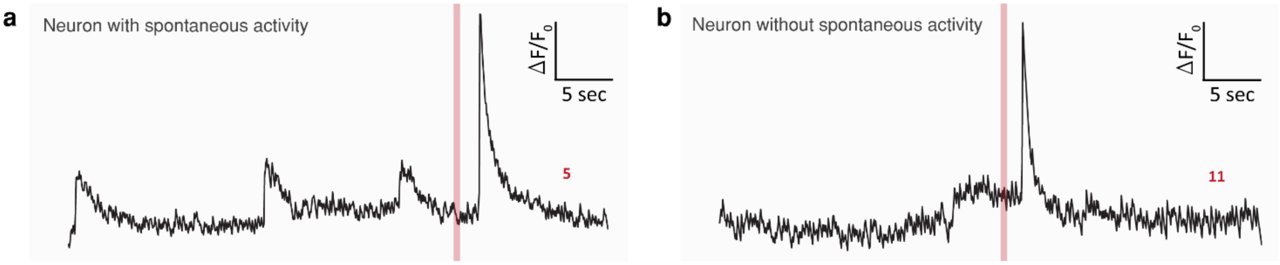

Supplementary Fig. 13 Baseline signals in Ca^{+2} imaging experiments. Representative quantification of calcium imaging for cultured primary neurons stimulated by PEMPs under FUS, with **a** and without **b** spontaneous electrical activity. PEMPs successfully stimulated cultured primary neurons in both cases. The red box represents the FUS excitation period.

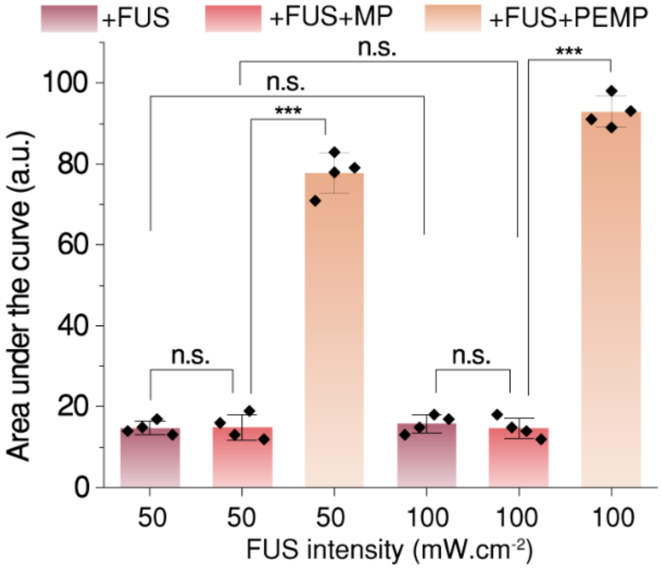

Supplementary Fig. 14 Control experiments on primary neurons under LIFU with and without magnetic Janus particles. Calcium responses of primary neurons without any particles, with magnetic Janus particles, and with PEMPs under 50 ms, 50, and 100 $mW.cm^{-2}$ FUS pulses ($n = 4$ independent experiments, each dot represents the mean of 10 individual neuron responses, two-sided t-test, $p < 0.001$). Data are presented as mean ± s.d.

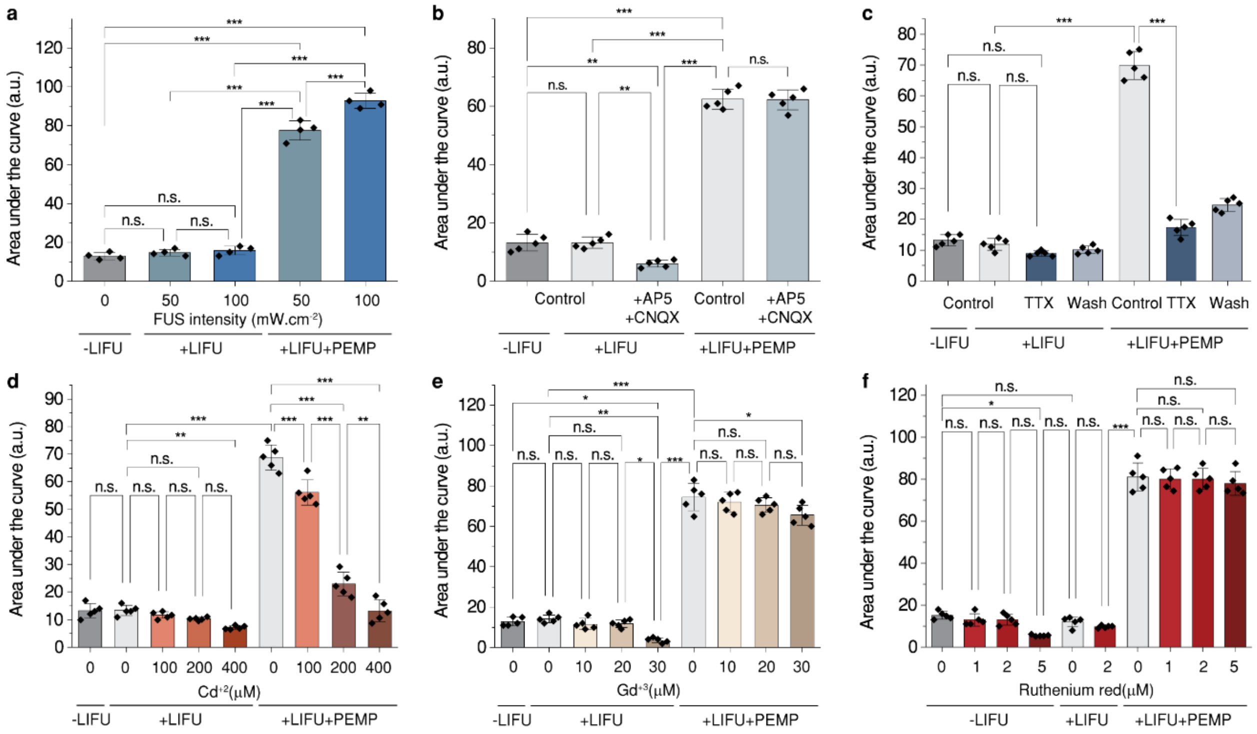

Supplementary Fig. 15 PEMP-induced neural stimulation under LIFU is mediated by the voltage-gated sodium and calcium channels. a Calcium response under different LIFU intensities with and without the PEMPs (n = 4 independent experiments for control and experimental groups.). **b** Calcium responses before and after treatment with synaptic blockers, AP5 and CNQX. **c** Calcium responses before, after, and washout treatment with TTX. **d** Calcium responses before and after treatment with Cd^{+2}. **e** Calcium responses before and after treatment with Gd^{+3}. **f** Calcium responses before and after treatment with Ruthenium Red. For all experiments in b, c, d, e, and f, n = 5 independent experiments for control and experimental groups. Bar graph values are represented as mean ± s.d.. Two-tailed, unpaired and paired T-tests were utilized for statistical analysis (*p <= 0.05, ** p <= 0.01, *** p <= 0.001).

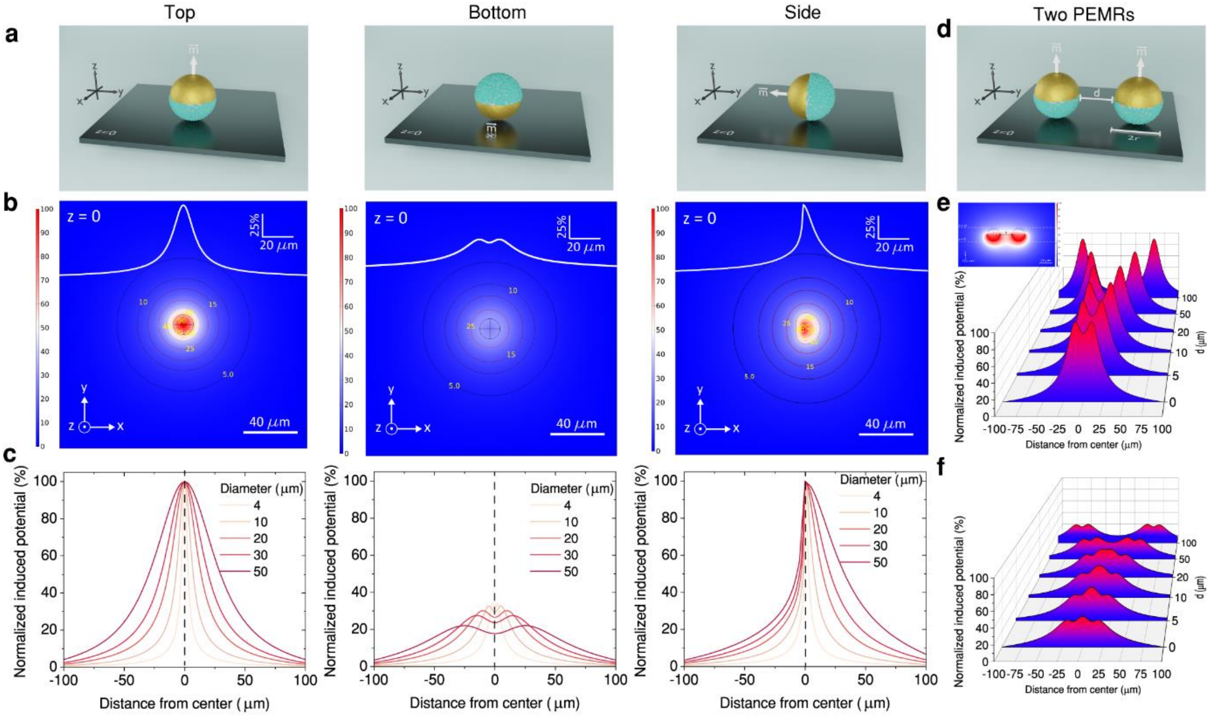

Supplementary Fig. 16 Spatial electric field distribution of PEMPs. a FEM simulation conditions for three different orientations of a single PEMP. Orientations were named with respect to magnetization direction as top, bottom, and side, respectively. **b** Normalized electrical potential distribution in the xy plane at z=0 for top, bottom, and side orientations, respectively. **c** Normalized electrical field decay in the xy plane at z=0 for different PEMP diameters. **d** FEM simulation condition for two PEMPs with 20 μm diameter, separated by distance d. **e** Electrical field decays in xy plane at z=0 and **f** z=2r for two PEMP conditions with changing separation distance d.

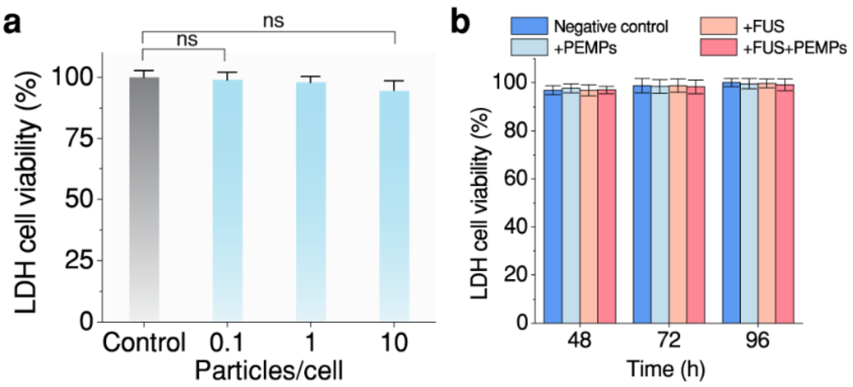

Supplementary Fig. 17 LDH cell viability results indicate high membrane integrity. a The effect of the PEMPs on the cellular membrane integrity of the differentiated SH-SY5Y cells for 0, 0.1, 1, and 10 particles/cell conditions assessed by the LDH assays. **b** LDH cell viability experiments of three experimental groups on differentiated SH-SY5Y cells for 96h in 1 particle/cell condition; under the excitation of FUS for 30 min with and without the PEMPs, and only PEMPs without the FUS excitation. All data are represented as mean ± s.d. Statistical significance is determined by two-sided Student's t-test and *$p < 0.05$ was considered statistically significant. ns, not significant.

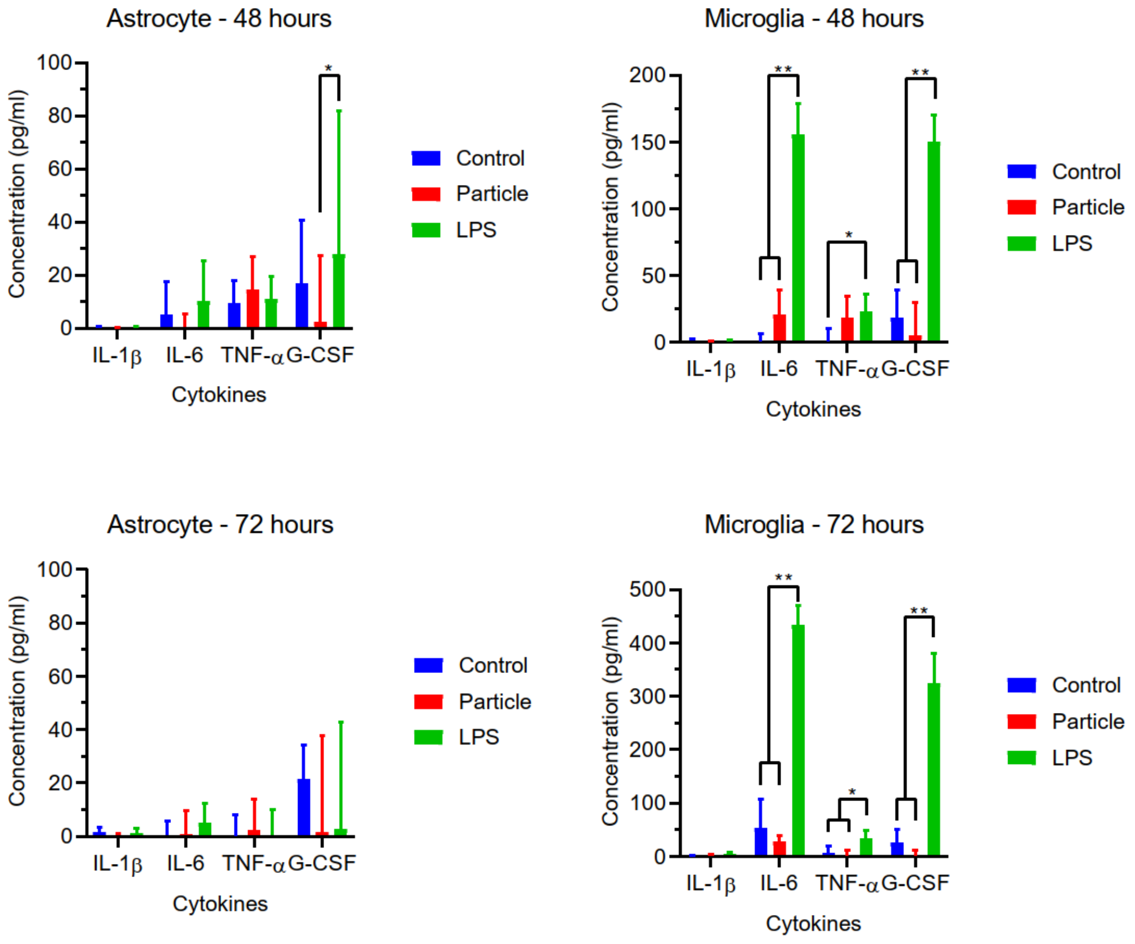

Supplementary Fig. 18 ELISA measurements for proinflammatory cytokines. The ELISA measurements were carried out after the incubation of PEMPs with astrocytes and microglia in various periods, 48 and 72 hours. There is no significant difference between the negative controls and the cells incubated with the particles for all proinflammatory cytokine measurements. PBS is used as a negative control and the bacterial lipopolysaccharide (LPS) is used as a positive control. n=12 for each experimental group; * indicates $p<0.01$ and ** indicates $p<0.001$.

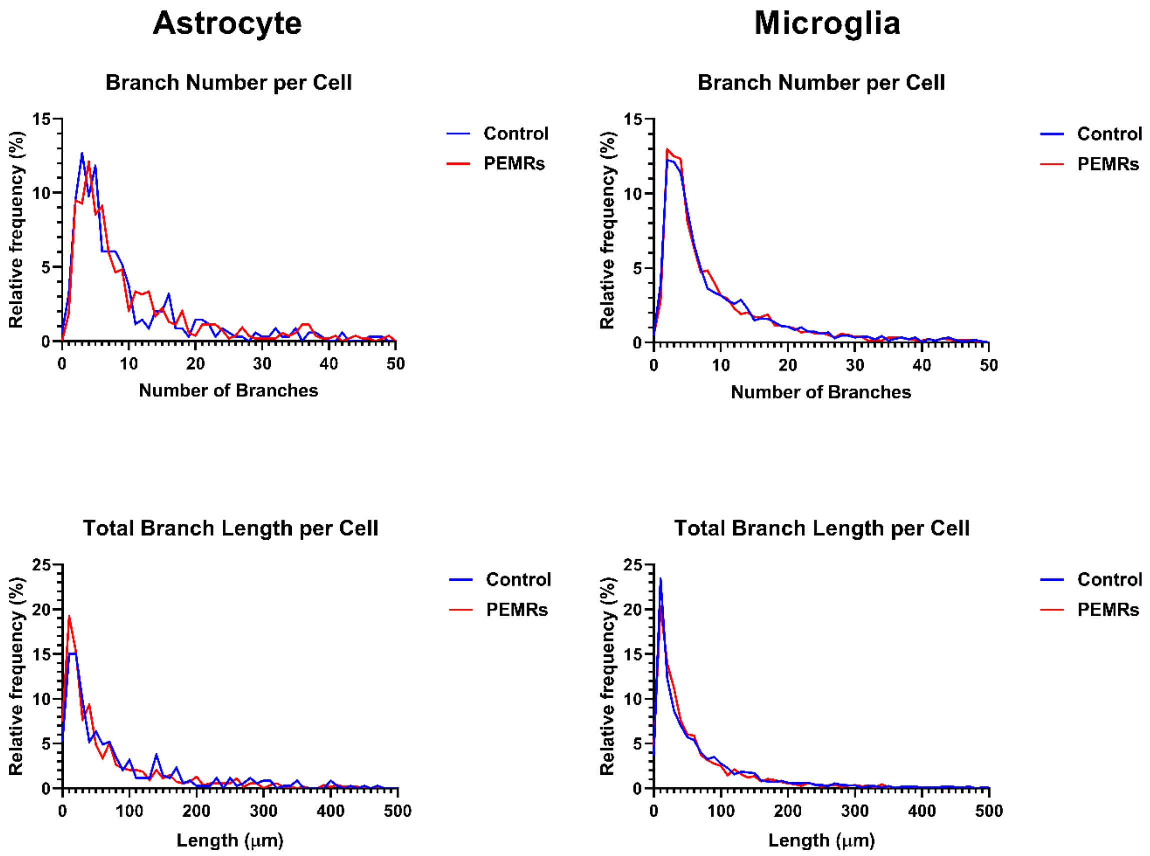

Supplementary Fig. 19 Morphometric analysis for astrocytes (left) and microglia (right). The number of branches and total length of branches for each cell were measured and calculated with automatic image processing software from immunofluorescence images of astrocytes and microglia after 72 hours of incubation with and without PEMPs. There is no statistically significant difference between the negative controls and the cells incubated with the particles for both the number and length of branches for each cell.

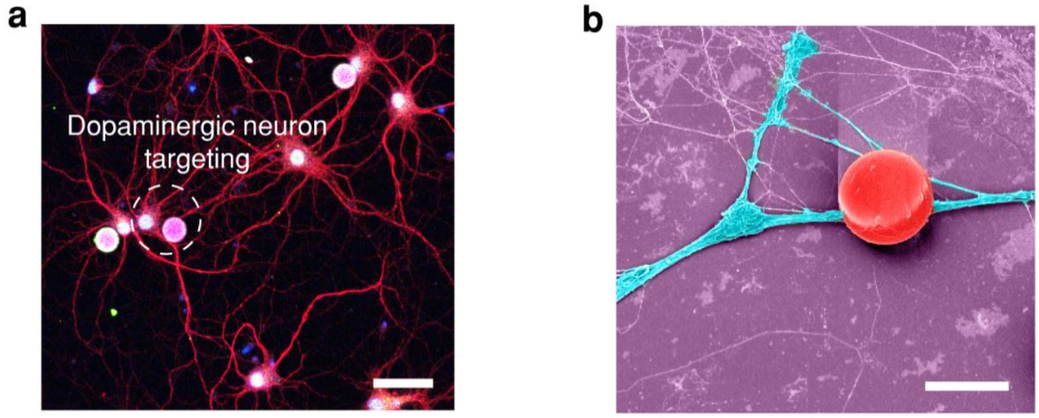

Supplementary Fig. 20 Dopaminergic neuron targeting of PEMPs. a Immunofluorescence image of PEMP attachment on dopaminergic primary neurons. Blue color, DAPI, expressed in the cell nuclei, green color, tyrosine hydroxylase, expressed in dopaminergic neuron cytoplasm, red color, MAP2 protein, expressed in the neural cytoskeleton, and magenta color indicates PEMPs. Scale bar, 50 μm. **b** Pseudo-colored scanning electron microscope image of a single PEMP in contact with a primary neuron. Scale bar, 20 μm.

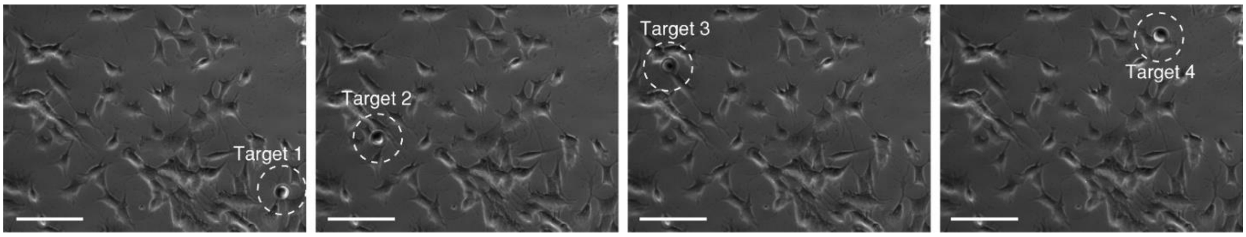

Supplementary Fig. 21 Demonstration of on-demand locomotion and stimulation capabilities. A single PEMP was magnetically actuated for four different target locations with the following trajectory indicated in Supplementary Video 1. Scale bar: 100 μm.

Supplementary Note 1. Hodgkin–Huxley (HH) model for potential build-up in the neural membrane due to piezoelectric charging of PEMP

To provide a perspective on piezoelectric neural stimulation, we built a numerical simulation[1,2] based on the Hodgkin–Huxley (HH) model[3]. Although this model only accounts for voltage-gated Na and K channels, it provides a base analysis for excitable cells. The numerical HH model simulations showed that, while a single charging/discharging cycle could not generate sufficient depolarization for neural activation due to the almost symmetric charging/discharging cycles of a piezoelectric sinusoidal signal by nature, multiple cycles provide the potential build-up on the membrane and could elicit action potentials[4] (Supplementary Fig. 4). These observations from the HH model could be attributed to the difference in the charging/discharging kinetics of the voltage-gated ion channels on the membrane. Mainly, in each sinusoidal cycle, membrane voltage increases during the charging period, while this potential increase does not fully decay in the discharging period due to the slower discharging kinetics of the neural membrane[4]. We utilized the discretization of the HH model described by Plonsey and Barr[2], where the HH equations for membrane potential were discretized in time for the numerical analysis (i.e. the membrane current, potential, and gating variables)[3]. This numerical simulation was implemented and run on MATLAB (version R2021b).

Supplementary Note 2. Induced momentarily stress on the cells due to the movement of the PEMPs on the neural cells

To quantify the induced force generated by the steering and locomotion of a single PEMP, we utilized the basic force balance of a microroller based on previous reports[5–7]. When a microroller moves on a planar surface, it creates a propulsion force to its translational direction, (F_P), and gravitational force (F_G) on the bottom wall[5–7]. If all the movement and forces were considered, the basic force balance of a microroller for F_P and F_G could be drawn as in Supplementary Fig. 22a.

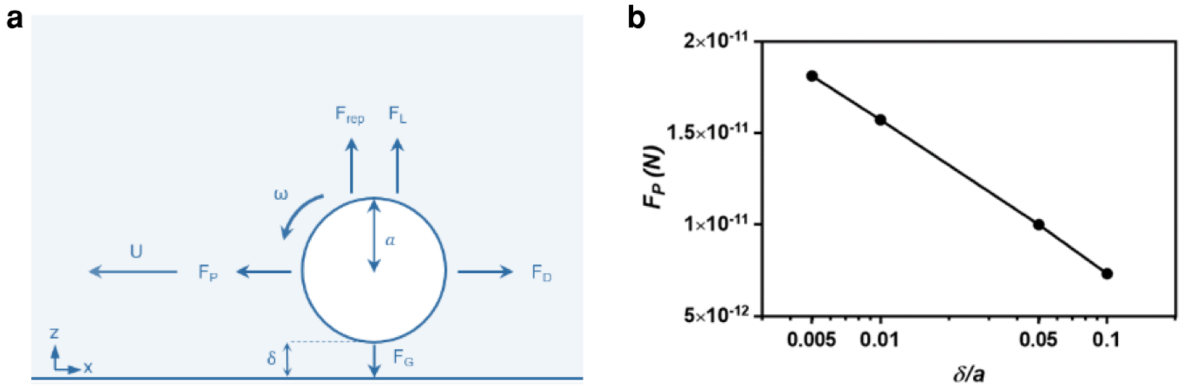

Supplementary Fig. 22. a Force balance on a microroller. **b** F_P produced by 20 µm microroller for different lubrication distances

The F_P and F_G are expressed as:

$$F_P = \pi\mu a^2\omega(\frac{4}{5}\ln\frac{a}{\delta} - 1.516) \quad (1)$$

$$F_G = \frac{4}{3}\pi(\rho_P - \rho_f)a^3 g \quad (2)$$

where μ dynamic viscosity of the fluid, a is the particle radius, ω is the angular velocity, δ is lubrication or separation distance, ρ_p the density of the particle, ρ_f the density of the fluid, and g is the gravitational constant. For a 20 µm microroller, which is our case for the PEMPs, the F_G forces were calculates as ~46.73 pN, and F_P for different lubrication distances were approximated as in Supplementary Fig. 22b.[5,8]

As a result, both the propulsion and gravitational forces are in the order of pN. With an exaggerated assumption of contact area is 1 µm^2, which would be much higher in the real case, the momentary stress applied by the microroller would be <50 Pa. In comparison, mechanical neural stimulation requires threshold force >200 nN and pressure value >5kPa via direct contact with atomic force microscopy cantilever[9]. Therefore, it is reasonable to assume that the movement of microrollers on biological cells would not induce potentially harmful effects or induce changes in the excitability of the neurons.

References

1. Schoen, I. & Fromherz, P. The mechanism of extracellular stimulation of nerve cells on an electrolyte-oxide-semiconductor capacitor. *Biophys. J.* **92**, 1096–1111 (2007).
2. Plonsey, R. & Barr, R. C. *Bioelectricity: A quantitative approach*. *Bioelectricity: A Quantitative Approach* (Springer US, 2007). doi:10.1007/978-0-387-48865-3.
3. Hodgkin, A. L. & Huxley, A. F. A quantitative description of membrane current and its application to conduction and excitation in nerve. *J. Physiol.* **117**, 500 (1952).
4. Howell, B., Medina, L. E. & Grill, W. M. Effects of frequency-dependent membrane capacitance on neural excitability. *J. Neural Eng.* **12**, (2015).
5. Bozuyuk, U. *et al.* Reduced rotational flows enable the translation of surface-rolling microrobots in confined spaces. *Nat. Commun.* **13**, 6289 (2022).
6. Bozuyuk, U., Alapan, Y., Aghakhani, A., Yunusa, M. & Sitti, M. Shape anisotropy-governed locomotion of surface microrollers on vessel-like microtopographies against physiological flows. *Proc. Natl. Acad. Sci. U. S. A.* **118**, 1–10 (2021).
7. Alapan, Y., Bozuyuk, U., Erkoc, P., Karacakol, A. C. & Sitti, M. Multifunctional surface microrollers for targeted cargo delivery in physiological blood flow. *Sci. Robot.* **5**, 1–11 (2020).
8. Bozuyuk, U., Yildiz, E., Han, M., Demir, S. O. & Sitti, M. Size-Dependent Locomotion Ability of Surface Microrollers on Physiologically Relevant Microtopographical Surfaces. *Small* **2303396**, 1–12 (2023).
9. Gaub, B. M. *et al.* Neurons differentiate magnitude and location of mechanical stimuli. *Proc. Natl. Acad. Sci. U. S. A.* **117**, 848–856 (2020).
