# Supplementary material for: Janus microparticles-based targeted and spatially-controlled piezoelectric neural stimulation via low-intensity focused ultrasound
Source: Nat Commun. 2024 Mar 5;15:2013. doi: 10.1038/s41467-024-46245-4 (PMC10915158; doi:10.1038/s41467-024-46245-4)
Supplement: Supplementary file 3 — Description of additional supplementary files [file 41467_2024_46245_MOESM3_ESM.pdf]

DESCRIPTION OF ADDITIONAL SUPPLEMENTARY FILES DOCUMENT

Supplementary Movie 1. Piezoelectric magnetic Janus microparticle (PEMP) locomotion and cell targeting. The PEMP was actuated using 10 mT, 5 Hz, out-of-plane rotating magnetic field to locomote the PEMP towards the targeted cells.

Supplementary Movie 2. Orientation control of piezoelectric magnetic Janus microparticle. The orientation of the PEMP towards the targeted cells was controlled using 10 mT, in-plane and out-of-plane rotating magnetic field at 0.1 and 0.5 Hz.

Supplementary Movie 3. 3D rendered fluorescent image of a primary hippocampal neuron without piezoelectric magnetic Janus microparticle (PEMP) attachment.

Supplementary Movie 4. 3D rendered fluorescent image of a dopaminergic neuron with piezoelectric magnetic Janus microparticle (PEMP) attachment.
