# Supplementary material for: Janus microparticles-based targeted and spatially-controlled piezoelectric neural stimulation via low-intensity focused ultrasound
Source: Nat Commun. 2024 Mar 5;15:2013. doi: 10.1038/s41467-024-46245-4 (PMC10915158; doi:10.1038/s41467-024-46245-4)
Supplement: Supplementary file 8 — Reporting Summary [file 41467_2024_46245_MOESM8_ESM.pdf]

nature portfolio

Corresponding author(s): Metin Sitti

Last updated by author(s): Jan 19, 2024

Reporting Summary

Nature Portfolio wishes to improve the reproducibility of the work that we publish. This form provides structure for consistency and transparency in reporting. For further information on Nature Portfolio policies, see our Editorial Policies and the Editorial Policy Checklist.

Statistics

For all statistical analyses, confirm that the following items are present in the figure legend, table legend, main text, or Methods section.

n/a	Confirmed	
☐	☒	The exact sample size (n) for each experimental group/condition, given as a discrete number and unit of measurement
☐	☒	A statement on whether measurements were taken from distinct samples or whether the same sample was measured repeatedly
☐	☒	The statistical test(s) used AND whether they are one- or two-sided<br>*Only common tests should be described solely by name; describe more complex techniques in the Methods section.*
☐	☒	A description of all covariates tested
☐	☒	A description of any assumptions or corrections, such as tests of normality and adjustment for multiple comparisons
☐	☒	A full description of the statistical parameters including central tendency (e.g. means) or other basic estimates (e.g. regression coefficient) AND variation (e.g. standard deviation) or associated estimates of uncertainty (e.g. confidence intervals)
☐	☒	For null hypothesis testing, the test statistic (e.g. F, t, r) with confidence intervals, effect sizes, degrees of freedom and P value noted<br>*Give P values as exact values whenever suitable.*
☒	☐	For Bayesian analysis, information on the choice of priors and Markov chain Monte Carlo settings
☒	☐	For hierarchical and complex designs, identification of the appropriate level for tests and full reporting of outcomes
☒	☐	Estimates of effect sizes (e.g. Cohen's d, Pearson's r), indicating how they were calculated

Our web collection on statistics for biologists contains articles on many of the points above.

Software and code

Policy information about availability of computer code

Data collection	Commercial software for data collection: Axoscope 11.2, Clampex 11.2, Zeiss Zenpro, COMSOL Multiphysics, ImageJ 1.54f, Leica LAS-X
Data analysis	Commercial software for data analysis: MATLAB R2021b, Origin 2021b, GraphPad Prism 9.0, ImageJ 1.54f, Microsoft Excell 2019

For manuscripts utilizing custom algorithms or software that are central to the research but not yet described in published literature, software must be made available to editors and reviewers. We strongly encourage code deposition in a community repository (e.g. GitHub). See the Nature Portfolio guidelines for submitting code & software for further information.

Data

Policy information about availability of data

All manuscripts must include a data availability statement. This statement should provide the following information, where applicable:

- Accession codes, unique identifiers, or web links for publicly available datasets
- A description of any restrictions on data availability
- For clinical datasets or third party data, please ensure that the statement adheres to our policy

All data supporting the findings of this study are available within the article and its supplementary files. Any additional requests for information can be directed to, and will be fulfilled by, the corresponding authors. Source data are provided with this paper.

Research involving human participants, their data, or biological material

Policy information about studies with human participants or human data. See also policy information about sex, gender (identity/presentation), and sexual orientation and race, ethnicity and racism.

Reporting on sex and gender	N/A
Reporting on race, ethnicity, or other socially relevant groupings	N/A
Population characteristics	N/A
Recruitment	N/A
Ethics oversight	N/A

Note that full information on the approval of the study protocol must also be provided in the manuscript.

Field-specific reporting

Please select the one below that is the best fit for your research. If you are not sure, read the appropriate sections before making your selection.

☒ Life sciences ☐ Behavioural & social sciences ☐ Ecological, evolutionary & environmental sciences

For a reference copy of the document with all sections, see nature.com/documents/nr-reporting-summary-flat.pdf

Life sciences study design

All studies must disclose on these points even when the disclosure is negative.

Sample size	Sample size of experiments was set to reach statistical significance of p = 0.05 at least. The preliminary power analyses conducted according to our group's previous study on neurostimulation. α-error = 0.01, β-error = 0.01, one-tailed used as power analysis parameters. The effect size of power analysis is calculated by using the cFOS staining and calcium imaging results from the reference publication: K. L. Kozielski et al., Nonresonant powering of injectable nanoelectrodes enables wireless deep brain stimulation in freely moving mice.Sci.Adv.7,eabc4189(2021). According to power analysis results, effective group size (n) is calculated as 6 (six) for each group to achieve statistical significance in electrophysiology studies. In our study, the minimum group size for the in vitro experiments is 6 (six) and is 3 (three) for all other experiments.
Data exclusions	No data were excluded.
Replication	All experiments were replicated at least three times, and each condition was analysed in triplicate. The experimental findings were reliably reproduced.
Randomization	Randomization is not applicable for the study.
Blinding	Blinding was applied to all experiments.

Reporting for specific materials, systems and methods

We require information from authors about some types of materials, experimental systems and methods used in many studies. Here, indicate whether each material, system or method listed is relevant to your study. If you are not sure if a list item applies to your research, read the appropriate section before selecting a response.

Materials & experimental systems

n/a	Involved in the study	
☐	☒	Antibodies
☐	☒	Eukaryotic cell lines
☒	☐	Palaeontology and archaeology
☒	☐	Animals and other organisms
☒	☐	Clinical data
☒	☐	Dual use research of concern
☒	☐	Plants

Methods

n/a	Involved in the study	
☒	☐	ChIP-seq
☒	☐	Flow cytometry
☒	☐	MRI-based neuroimaging

Antibodies

Antibodies used	DAPI-supplemented mounting medium (ab104139, Abcam), MAP2 (ab5392, Abcam), tyrosine hydroxylase (ab137869, Abcam), S100b (ab52642, Abcam), Iba1 (ab178846, Abcam), anti-chicken Alexa Fluor 555 (A-21437, Invitrogen), anti-rabbit Alexa Fluor 488(A-11008, Invitrogen), and anti-mouse Alexa Fluor 647 (A-21235, Invitrogen)
Validation	All validation data of antibodies are available in manufacturer's websites:<br>DAPI Supplemented Mounting Medium (ab104139, Abcam, Cambridge, UK); https://www.abcam.com/mounting-medium-with-dapiaqueous-fluoroshield-ab104139.html<br>MAP2 (ab5392, Abcam); https://www.abcam.com/products/primary-antibodies/map2-antibody-ab5392.html<br>tyrosine hydroxylase (ab137869, Abcam); https://www.abcam.com/products/primary-antibodies/tyrosine-hydroxylase-antibody-ep1532y-neuronal-marker-ab137869.html<br>S100b (ab52642, Abcam); https://www.abcam.com/products/primary-antibodies/s100-beta-antibody-ep1576y-astrocyte-marker-ab52642.html<br>Iba1 (ab178846, Abcam); https://www.abcam.com/products/primary-antibodies/iba1-antibody-epr16588-ab178846.html<br>anti-chicken Alexa Fluor 555 (A-21437, Invitrogen); https://www.thermofisher.com/antibody/product/Goat-anti-Chicken-IgY-H-L-Secondary-Antibody-Polyclonal/A-21437<br>anti-rabbit Alexa Fluor 488(A-11008, Invitrogen);https://www.thermofisher.com/antibody/product/Goat-anti-Rabbit-IgG-H-L-Cross-Adsorbed-Secondary-Antibody-Polyclonal/A-11008<br>Anti-mouse Alexa Fluor 647 (A-21235, Invitrogen); https://www.thermofisher.com/antibody/product/Goat-anti-Mouse-IgG-H-L-Cross-Adsorbed-Secondary-Antibody-Polyclonal/A-21235

Eukaryotic cell lines

Policy information about cell lines and Sex and Gender in Research

Cell line source(s)	All cell lines used in this study were purchased from commercial manufacturers. The SH-SY5Y cell line from DSMZ (German Collection of Microorganisms and Cell Cultures, DSMZ No.: ACC 209), Primary rat hippocampal neurons from Thermo Fisher Scientific (Gibco™, A1084101), C8-D1A cells (CRL-2541), EOC13.31 cells (CRL-2468), and LADMAC cells (CRL-2420) from ATCC (American Type Culture Collection) were purchased.
Authentication	All cell lines were purchased from commercial manufacturers; due to that, no additional authentication protocol was performed.
Mycoplasma contamination	All cell lines were tested for mycoplasma contamination. No mycoplasma contamination was found.
Commonly misidentified lines (See ICLAC register)	No commonly misidentified cell lines were used.
